# Unveiling the Impact of Psychological Traits on Innovative Financial Decision-making in Small Tourism Businesses

**DOI:** 10.1007/s13132-022-00987-y

**Published:** 2022-02-27

**Authors:** Navjot Sandhu, Hatem El-Gohary

**Affiliations:** 1 Birmingham City University Business School, BLSS, Birmingham City University, Birmingham, UK; 2 College of Business & Economics, Qatar University, Doha, Qatar

**Keywords:** Innovative financial decision-making, Entrepreneurs, Financing preferences, Personal finance, SMEs, India, Tourism firms, Theory of planned behaviour, Family firms

## Abstract

Understanding factors affecting innovative financial decision-making involves the usage of more than one theory-driven model including those related to psychological behaviour (e.g. theory of planned behaviour). This study validates a conceptual framework extending the traditional financing model (TFM) and behavioural financial decision (BFD) to explain innovative financial decision-making utilising both qualitative and quantitative approaches. Based on a sample of 140 small tourism businesses, employing structured equation modelling, the findings reinforce the importance of the behavioural approach on innovative financial decision-making in small tourism businesses. The findings does not only make a respectable contribution the field, but also offer a practical evidence for the adaptation of the theory of planned behaviour in developing countries.

## Introduction

Tourism make a great economic impact on any country GDP and economy in general (Fu et al., 2020; Sánchez-Medina et al., 2020; El-Gohary, 2012; Ohlan, 2017; García-Villaverde et al., 2017; Tugcu, 2014; Chou, 2013; El-Gohary, 2020; Balli et al., 2015; Sirgy, 2019; El-Gohary & Eid, 2012). Recent studies suggest that the contribution of tourism industry in global GDP increased to 8.5% and generated 2.5 million jobs in 2016 and by 2024 would have an increase of 2.0% (Motta & Sharma, 2020). Meanwhile, tourism in most countries is dominated by small- and medium-sized enterprises (SMEs).

Most of the published work within the field highlighted the importance of SME contributions to economic growth, innovation, poverty relief, and employment growth worldwide. The works of Rayappa and Arora (2021), Weqar et al. (2021), Wu and Wu (2020), Sirgy (2019), Dosumu et al. (2017), Zaki et al. (2021), Du et al. (2015), Ramaswamy (2014), Cernat and Gourdon (2012), Beck et al. (2011), Kalhoro et al. (2011), Storey and Greene (2010), European Union (2009), Storey (1994), Hamad et al. (2018), and Shah et al. (2015) are just few examples of such published work. However, by looking at the literature, it is noticed that most of the literature focused on other industries and not on tourism (Jaafar et al., 2011), whereas it managed to gain attention as an effective tool to initiate progress in developed countries (UK, USA, etc.) as argued by El-Gohary et al. (2018), Schubert et al. (2011), and Ateljevic (2007). However, as the academic fraternity come to acknowledge the importance of tourism and its impact on developing countries ability to gain hard currency and improve unemployment rates, small tourism businesses started to gain attention within the literature in the last few decades. (Motta & Sharma, 2020; Li et al., 2016; Chou, 2013; Thomas et al., 2011; Thomas & Augustyn, 2007; Hall & Page, 2009; El-Gohary & Eid, 2014). Nonetheless, topics linked specifically to tourist companies in developing countries are still less researched and presented in the literature (Nemasetoni & Rogerson, 2005).

Most tourism companies in less developed countries face many serious difficulties that are generated mainly from the market that these companies operates in, which is immature and characterised by its undeveloped financial infrastructure and environment. More specifically, access to finance by tourism companies (including tourism SMEs) remains a major challenge (Hussain et al., 2020; Beck et al., 2008, 2011, 2006, 2005; Berger & Udell, 2006). In contrast, tourism companies in developed economies have better markets, better financial systems, good infrastructure, and easy access to finance as well as other resources (Britton, 1982; Salimullah, 2021; Tleuberdinova et al., 2021).

Many studies (e.g. Rayappa and Arora (2021), Hussain et al., 2020; Thomas et al., 2011; Hussain et al., 2006; Beck et al., 2008, 2011, 2006, 2005) looked at the financial restraints faced by SMEs in both developing and developed nations. However, scholar like Lynch (1998) and Thomas et al. (2011) argues that research related to SMEs in general cannot be applied to SMEs operating in all sectors. The authors argue that Lynch (1998) and Thomas et al. (2011) viewpoint is not valid as SMEs are characterised to be heterogeneous. Additionally, the same group of factors affects the performance of SMEs regardless of its industry. From the authors point of view, the vital flaws of the prevailing published research work are the fact that there was no main or fundamental change in scholars views on tourism SMEs (Thomas et al., 2011). Small tourism companies have their unique features, and academic studies have failed to differentiate between different subsectors (Thomas et al., 2011; Bridge et al., 2008; Smith, 2006). For instance, tourism companies are heavily reliant on technology and well-developed tourism companies can have access to international tourism markets (Kim, 2006).

Meanwhile, when considering a main developing tourism SME market like India, it is noticed that Indian middle class is driving the growth in the Indian tourism industry. Along with various other Indian governmental initiatives such as visa on arrival for the nationals of some countries, tax exemptions for tourism, and allied businesses, campaigns ‘Incredible India’ and ‘Colours of India’ to promote Indian culture and diversity (Indian Chamber of Commerce, 2019; Singh & Turan, 2007) led to a good growth in Indian tourism industry. Increased domestic and foreign international tourism has generated numerous jobs in many Indian economic sectors. Additionally, according to available statistics, tourism industry in India employs about 20 million individuals (Indian Chamber of Commerce, 2019; UKEssays, 2018). Due to the availability of more educational resources now in India and cordial nature of its population, who are very enterprising, tourism-related businesses are booming, hence, increasing number of hotels, travel agents, and web portals being opened in various regions across India (UKEssays, 2018). The culture of tourism of India is built on seven main patriotic principles and values; these principles are as follows:

- Swaagat (welcome),
- Soochanaa (information),
- Sanrachannaa (infrastructure),
- Suvidha (facilitation),
- Sahyog (cooperation),
- Safaai (cleanliness), and
- Surakshaa (security) (Indian Chamber of Commerce, 2019; Singh & Turan, 2007).

Therefore, in order to maintain standards and provide quality services, small tourism companies need funds. Hence, there is finance gap for small tourism businesses, which can hinder its progress. Of course, COVID-19 made the situation worse for small tourism businesses. However, with the availability of COVID-19 vaccines, it is hoped that the pandemic will end soon which offer good potential for economic recovery and growth. During this post-COVID-19 era of opportunities and growth potentials for tourism industry, where behavioural factors are superseding economic theories, it becomes necessary to explore how these small tourism companies are funding their operations. This should include the investigation of the financing choices made by owners or the managers of the small tourism companies.

In contrast, entrepreneurship and tourism studies are limited in emerging economies. Little research of this nature has been done in the Indian context, especially for small tourism firms. There is no comprehensive empirical research especially for small tourism businesses that has considered how a firm’s individual features (such as firm time in business, the size of the company, and the owner or manager behaviour) affect both its capital structure as well as financing decisions. Given these points, this is an important study to emphasis upon this relatively under researched area or section of the small business population by identifying uncharted factors affecting small tourism businesses, incorporating insights from social psychology with financial choices/decisions made by owners or the managers of the small tourism companies. Such research is important as failure to recognise the influence of behaviour could lead to various misinterpretations such as lack of financial support from the supply side.

To address these issues, this research applies the Ajzen (1991) Theory of Planned Behaviour to inspect the impact of owner/managers behaviours, norms, attitudes, and motivations on the financing choices made by them. Consequently, the current research aims to use Ajzen (1991) theory to develop and test an extended model to:

- Investigate the effect of owner and/or managers’ behaviour on financing decisions.
- Examine the interrelationship among family norms and motivating factors and examine the relative strength of family norms on attitude and behavioural intentions towards using particular sources of finance in a country like India where norms are anticipated to have greater strength compared to attitude.

Although the finance gap for SMEs is widely acknowledged in both developed and developing countries, it will be wise to investigate issues influencing financing choices from the demand side as well as exhausting the theory of planned behaviour.

## Review of the Literature, Theory, Hypotheses Development, and Conceptual Framework

### Financing of Small Tourism Companies

Academic fraternity have recognised that financing can be an impediment for small tourism businesses owner/managers to realise their desire of starting and operating a successful business (King et al., 2001; Morrison & Teixeira, 2004; Sandhu & Hussain, 2017). There are presumptions that tourism business requires little capital. Nevertheless, such assumptions are not true in many cases (such as the case of tourism companies dealing with tourists from high-income or highly developed nations). Such tourists tend to require the availability of advanced facilities and infrastructure, which tend to cost a lot of money (Badulescu et al., 2015; Page et al., 1999; Sandhu & Hussain, 2015; Morrison & Teixeira, 2004). Resultantly, in reality, it is pretty difficult to maintain a conducive milieu or small tourism business infrastructure due to limited access to relevant resources.

Lack of accessibility to suitable finance for small tourism businesses is repeatedly one of the causes preventing small tourism businesses and entrepreneurs from pursuing ground-breaking and novel business products or ideas/plans. Insufficient finance is expected to limit small tourism business growth potential, which can consequently lead to increase small tourism business failure rates especially for start-ups (Carlislie et al., 2013). Meanwhile, small tourism businesses have unique features and suffer from finance gap(s) and limited access to relevant resources, which make it challenging for such companies to set up and/or maintain a conducive environment (Kumar & Rao, 2016). Nevertheless, small tourism businesses (despite their unique characteristic) require exploration to face the explicit challenges facing it to obtain sufficient finance and design tools and approaches to boost growth. Tourism companies with acceptable funds incline to be more robust to its innovation (Thomas et al., 2011; Grindley & Teece, 1997). Nevertheless, it is vital to understand that financial constraints (e.g. lack of or limited collaterals and weak past credit history) hinder small tourism business prospective. Given the characteristics of the tourism companies in India, financial constraints can affect small tourism companies’ success and/or survival severely. In consideration of the above, the authors propose the following hypotheses:

- **H1**: Access to finance is positively associated with small tourism businesses’ growth and development.
- **H2**: Decline in the importance of collateral with the age of the business improve has a positive influence on the small tourism businesses’ growth.

Among the difficulties that small tourism businesses face is the need for financial resources as a preceding assurance to protect their business. In most cases, creditors have to rely on small tourism businesses’ anticipated cash flows to appraise their business plans. However, the Indian small tourism businesses/SMEs rely on social capital such as relationship lending and the dense networks of external and internal influences to alleviate information asymmetry and augment firms’ access to information (Sandhu & Hussain, 2017; Sandhu et al., 2015). Considering the counterbalancing forces noted above, the authors propose the following hypotheses:

- **H3**: Increased external influences lead to easy access to finance or loan acceptance of SMEs.
- **H4**: Internal influences have positive effects on access to finance.

### Economic Perspective

#### Conventional Financing Theories of Decision-making

When companies conduct decision-making process related to the type of capital-structure they wish to accomplish, they depend on some theories to help them in making the right decision. Among such theories:

- The Static Trade Off Theory (SToT), and
- The Pecking Order Theory (POT).

The first theory illustrates that companies need to get a debt-equity ratio considering cost minimisation and aiming to make their debt choices to minimise the cost, avail themselves of tax benefits, and reduce agency costs (Park & Jang, 2018; Graham & Harvey, 2001; Myers & Majluf, 1984; Modigliani & Miller, 1963). These theories assumed that investors behave rationally. Nevertheless, that is not always the case, as Barton and Gordon (1988) showed in highlighting the irrational elements of decision-making.

However, the encounters faced by SMEs in general are also applicable to small tourism companies and the pattern of financing choices for tourism firms are in line with the second theory—POT (Holmes & Kent, 1991; Bruno & Tyebjee, 1985; Myers & Majluf, 1984). Due to small tourism companies’ opaqueness like other SMEs, small tourism businesses also incur information costs and agency costs associated with debt (Psillaki, 1995; Ang, 1992). Thus, to evade the information and other associated costs, small firms favour to use internal finance beforehand looking for external debt. Small firms give preference to debt then equity as it aids in maintaining control over the business and evades interference in management decisions. Consistently, unlike big companies, small tourism business owners or managers incline to use their internal funds. Even when they need extra funding, they obtain it only from banks. As a result of this, most small tourism companies’ capital structure tends to be from owners’ resources and away from depending on debt capital. However, in reality, due to small tourism business owner managers’ financial awareness and literacy, their over reliance on external funding outweighs their reliance on interior funding. Over the years as the business matures, it enables small tourism business owner and managers to prepare better financial information, resultantly, proper trading records accomplishments and explore better finance provisions (Sandhu & Hussain, 2017). Resultantly, the authors propose the subsequent hypothesis:

- **H5**: Changes in the financing preferences of the small tourism businesses owner or managers influences positively small tourism businesses availability of finance and growth.

Researchers argue that SToT and POT theories might not be pertinent to firms in developing countries because of institutional and cultural alterations (Zellweger et al., 2019; Sarria-Allende et al., 2006; Morrison & Teixeira, 2004). Nonetheless, these theories could not describe SME financing preferences and decisions in developing economies because of the different sociological and psychological paradigm and various other types of obstacles faced by SME owner/managers (Baixauli-Soler et al., 2021; Kim et al., 2021; Weqar et al., 2021; Zellweger et al., 2019; Newman et al., 2011). Still, most small and medium enterprises employ elements of traditional capital theories (Kieschnick & Moussawi, 2017). However, these traditional theoretical approaches fail to rationalise those financial decisions, besides capturing the need of collateral; hence, very little is known about why small and medium enterprises employ certain forms of financing. As the owner and managers’ unique personal characteristics such as attitude, observed norms, and control have an important impact on the company financial decision-making (Koropp et al., 2014), it becomes necessary to explicate the heterogeneity of the process of financial decision-making by using Ajzen’s theory. This theory has become increasingly popular since the late 1990s in explaining business ethics, consumer decisions, and entrepreneurial intentions/behaviour (Kuiken, 2015). The purpose of the theory of planned behaviour (ToPB) is to envisage and designate the behaviour in an explicit setting.

### Extension of ToPB for Financing Decision-making in Small Tourism Businesses

**Fig. 1:**
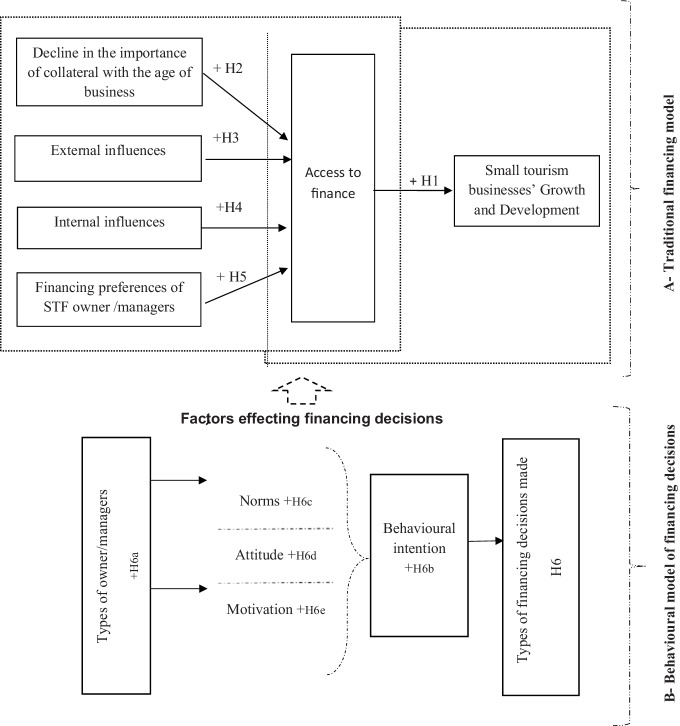
Summary of conceptual models of financing decision (BFD)-making in small tourism businesses (extended theory of planned behaviour for decision-making)

Previous studies have used the ToPB in several settings, such as to explain entrepreneurial success, failure, behaviour, and intentions (Gjergji et al., 2022; Baixauli-Soler et al., 2021; Schuckert et al., 2018; Hsu et al., 2017; Delmar & Wiklund, 2008; Krueger et al., 2000; Stavrov, 1998) and successors’ intentions in family organisations (Baixauli-Soler et al., 2021; Sharma et al., 2003, 2005). According to this theory, decisions-makers’ behavioural intentions determine their immediate (proximate) behaviour and decisions (Ajzen, 1987, 1991). The theory of planned behaviour is an extension of the theory of reasoned action that introduces the concept of behavioural control as an additional determinant of intention and behaviour (Ajzen & Schmidt, 2020; Conner, 2020; Ferreira et al., 2021; Lim & Weissmann, 2021). Hence, ToPB is drawn upon the notion that people behave in a functional way that they consider the available opportunities and resources and explicitly or implicitly take account of the implications of their actions. Therefore, behavioural intention arises from an individual’s characteristics such as attitudes, values, standards, beliefs, and behavioural control. This study considered the following descriptors to describe the main components of extended ToPB model:

As mentioned earlier, research in various perspectives strongly supports the academic relationships identified among attitudes, intentions, and behaviours (Koropp et al., 2014). For this paper, the ToPB has been adopted to investigate whether financing choices adopted in small tourism businesses might be a consequence of individual attitudes of the owner and/or managers. The study of Koropp et al. (2014) managed to inspect the bearing of owner/managers’ behaviour on financial decisions for German family businesses; however, no research has sought to differentiate the financial decisions taken by entrepreneurs and family firm owner/managers and non-family firms to run their businesses in general and India-based small tourism businesses specifically. Hence, the next set of hypotheses (presented in section B of Fig. 1) posed are as follows:

- **H6**: Differences in financing choices made by entrepreneurs, owner, or managers of family owned and nonfamily small tourism businesses are affected by individual attitudes of the owner and/or managers.
-
  - **H6a: **Type of firm ownership has an effect on the financing decisions made by the owner and/or managers.
  - **H6b**: Behaviour of owner and/or managers affects their financing decisions.
  - **H6c**: Norms of the companies have an effect on owner and/or managers’ financing decisions.
  - **H6d**: Attitude of owner and/or managers has an effect on the type of financing used by them.
  - **H6e**: Motivation of owner and/or managers has an effect on their financing choices.

### Conceptual Framework

The studies mentioned above provided an insight on the contribution of small corporations (working in tourism sector) towards the economy at large. The conclusion reached by Hussain and Matlay (2007), which is also applicable to Indian SMEs, is that limited access to finance constricts their growth. The abovementioned studies provide insight into the nature of the obstacles faced by SMEs at large, nonetheless lack of exhaustive research studies with policy recommendations to propose effective solutions in order to overcome the financing restraints confronted by Indian small and medium tourism firms in general, and in Punjab specifically. Financial institutions can make an energetic contribution to the growth and expansion of tourism industry because access to finance is necessary for the uplift of a state’s tourism industry in all domains. In developing countries, availability of finance can exert some influence on the growth and progress of SMEs in general and small tourism businesses specifically. However, the tentative model (Fig. 1) is developed and derived from relevant literature on the importance of collateral (Hussain et al., 2006; Sandhu et al., 2012a, b); financing preferences of owner managers (Sandhu et al., 2015); role of external and internal influences (Sandhu, 2007; Sandhu & Hussain, 2015; Sandhu et al., 2017). Section A of the Fig. 1 indicates the relationships between access to finance and small tourism businesses’ development. This model was designed to exhibit that real world is multifaceted and conservative finance models have failed to capture the operational dynamics that can allow small tourism businesses to understand and appreciate their full potential. Section B of the framework elucidates the heterogeneity of financial decision-making. The model supposes that external and internal influences increase access to formal finance. Moreover, it divulges that the financing choices of the small tourism businesses’ owner/managers change over time, which will have a positive influence on small tourism corporations’ growth and expansion.

## Methodology

To pursue the research objectives, this study integrates both qualitative and quantitative measures. Any research approach runs some risk of being bias-affected (Scandura & Williams, 2000). In order to reduce such negative effects as errors due to systematic and probabilities planning, a triangulation approach was adopted. Qualitative research is pertinent in exploring and understanding developments, concurrently, permitting answering both the ‘why’ and ‘how’ questions related to the research relationships, which are otherwise too problematic to answer using other methods (e.g. quantitative research methods) as explained by Reay (2014). Utilising interviews, case studies, or observations enables researchers to vastly expand their understanding on why some financing sources are preferable used by small firms (Koropp et al., 2014; Langley & Abdallah, 2011).

Built on a thorough evaluation of other research studies on financing of SMEs (for example Hussain & Matlay, 2007; Hussain et al., 2006), a survey questionnaire was developed. This questionnaire was designed with special attention to obtain quantitative as well as qualitative information on the actual and preferred sources of finance accessed by the participants and perceived or actual barriers experienced by SMEs’ owner/managers during various stages of the business cycle in Indian Punjab. The questionnaire was first distributed to SMEs in Indian Punjab in 2013 to investigate the financing constraints and growth of SMEs in Indian Punjab. However, financing behaviour in small tourism businesses is not utterly focused by financial causes, but also by non-economic concerns (e.g. risk-acceptance and captivating tendency, emotions, business goals, preferred values). Hence, traditional theories are unable to entirely clarify financing behaviour within these small and medium enterprises. Furthermore, it is worth inspecting how behavioural arguments explain financing behaviour.

Hence, the initial questionnaire was revisited and modified to fill up literature gaps that links such non-economic concerns (e.g. the business goals, motives, and attitudes of owner/managers). A rigorous pilot test was conducted among five tourism companies in Delhi. However, for refinement, validity of the questionnaire was checked to achieve pre-defined magnitudes successfully. Finally, a self-administered structured questionnaire was designed based on collective inputs from the pilot study to measure four variables representing the owner/managers’ characteristics. Each variable was measured using a five-point Likert scale developed specifically for this study. Moreover, to obtain deeper understandings of the examined phenomena, a grounded theory multi-respondent approach was used. Therefore, everyone who was involved in the process of financial decision-making (either directly or indirectly) was probed. Grounded theory expedites amplification of theoretical concepts (Glaser, 2004). Nevertheless, for this study, qualitative method provided flexibility, to fully apprehend the meanings and views expressed by the respondents (Miller & Salkind, 2002). Moreover, this permitted the researcher to warrant the representativeness of the study by establishing a consensus-based dataset in which method biases were abridged (Holt et al., 2017; Podsakoff et al., 2003).

The data were collected in Punjab and Delhi using a non-probability (purposive) snowball sampling method, which allowed for choosing the respondents on the basis of preordained wisdom and where owner/managers referred other tourism companies. Efforts were made to ensure representation of a variety of SMEs from the tourism sector operating hotels, planning or arranging trips, booking domestic and international flights, etc. A total of 300 questionnaires were directed to selected tourism companies from Punjab and Delhi; however, when the contacts were made, only 220 were willing to participate, leaving a sample of 220 viable firms. In order to reduce non-response bias, numerous follow-up reminders were made. In the end, 140 valid responses were reverted, giving a response rate of 46%, whereas 80 responses were discarded due to lack of full information. Therefore, to safeguard the survey responses’ validity and its representativeness of the wider population, a nonresponse bias examination was conducted to parallel early responses and late responses. *χ*^2^ test results indicated no substantial difference among the two groups of participants (at the 5% level of significance) suggesting that the data is clear from non-response bias. Normally, the response rate is 20–25%, so a range of techniques are used to improve this.

Of the 140 respondents who responded to the questionnaires, fifteen were selected as the most appropriate persons to be interviewed. Therefore, fifteen face-to-face were semi-structured in depth interviews. Each interview lasted for 30–50 min and was carried out with STF owner/managers who were willing to cooperate. Professional transcriptionists documented all interviews. Afterward, the data were coded and analysed depending on Nvivo S/W (a program that can support in classifying and plotting patterns in qualitative data). Moreover, the information was collected from various other portals and means such as internet, pamphlets, and observations by the researchers during the conduct of the survey. These techniques made it possible to access small and medium tourism enterprises in Punjab and Delhi. Almost all the STF owner/managers (participants) could read and write English; hence, there was no need to translate the survey into either Punjabi or Hindi, however, when needed; one of the researchers was available for translation. The anonymity of the participants was guaranteed. Thus, all the individuals who were approached were confirmed that confidentiality would be strictly maintained. It was also clearly indicated at the beginning of the questionnaire that respondents have full freedom to express their own experiences and views. In the hindsight, it helped to lessen the level of fear and anxiety amongst the participants and encouraged them to speak out the facts rather than providing socially desirable replies (see also Chang et al., 2010).

### Reliability and Validity Analysis

Cronbach’s alpha was conducted to test the validity and reliability of all constructs prior to undertaking detailed analysis (Tavakol & Dennick, 2011; Babbie & Mouton, 2002). The results are shown in Table 1. Hair et al. (1998), El-Gohary (2012), and Cooper and Schindler (2008) illustrated that the cut-off score for acceptable alpha is 0.7; however, some other scholars depend on lower alpha values (Griffee, 2012; Shay, 2008). The results exposed that all the research variables had a high reliability alpha ranging from 0.862 to 0.868 and a great item-to-item correlation, stretching from 0.585 to 0.695. Hence, the value of both of them is significant (Gliem & Gliem, 2003; Tavakol & Dennick, 2011). Most items appeared to be worthy of retention, since if deleted it will lead to decrease in alpha. Therefore, the used research constructs are satisfactorily suitable for directing further data analysis.

**Table 1:** The components of extended ToPB

***Owner managers/firm ownership***	These small tourism businesses are family and non-family companies. Hence, it will be interesting to investigate whether the financial decisions made by family owned owner/managers are different from non-family owned firms
***Behaviour intention***	Arises from individual’s characteristics such as attitudes, values, standards, beliefs, and behavioural control whether to use particular source of finance or not. Therefore, it will be interesting to investigate whether behaviour could or could not affect financing decisions
***Norms***	Family norms, social norms, individual’s (owner/managers) recognition of the social compression to achieve or not to achieve the behaviour under consideration. Therefore, norms could or could not affect financing decisions
***Attitude***	The judgemental decision is influenced by the owner/managers’ interpretation of opportunities and risk attitude (risk takers, risk neutral, risk averse). Attitude could or could not affect financing decisions
***Motivation/perceived behavioural control***	Skills, knowledge, other external, and internal factors that affect owner managers’ decision-making; possessing the capabilities that can modify an individual’s belief in the likelihood of completing the specific tasks

Furthermore, since the data for the research variables (financing choices/managers’ characteristics) were collected using the identical survey instrument at the same time from the same respondents, there was a danger that the data could be prone to common method bias as explained and argued by Chang et al. (2010). Therefore, Harman’s one-factor test was conducted to study the presence of such bias by conducting an exploratory factor analysis (Scott & Bruce, 1994; Podsakoff & Organ, 1986). The test results indicated that items were not concerted in one factor. Instead, they were loaded into more than one factor. In addition, the single-factor structure was unable to elucidate a substantial covariance (35.27%) in both dependent and the independent variables. All factors were extracted with eigenvalues greater than one, with no single factor explaining a mainstream of the variance. Conclusively, based on multiple approaches and evaluations, this is evident that the data is clear from common method. Since numerous issues/factors were identified rather than one single factor.

### Empirical Model

The data collected were also quantitative in nature, and the study sought to conclude the grade of connotation among different variables. Thus, the descriptive and inferential instruments of correlation and multiple regressions were applied. Correlation analysis was conducted to test variables’ association with each other, while regression analysis was used to investigate the hypothetical relationships among different research variables. To analyse the data, the following regression model was used:

$$y_{it}=a+{\beta^t}_1{X^t}_1+{\beta^t}_2{X^t}_2+\dots{\beta^t}_n{X^t}_n+\it\EUR_{it}$$

where:

- *y*_*it*_ specifies the dependent variable.
- *X*_*it*_ as a function of STF characteristics owner/managers’ characteristics (the slope representing the degree of change in independent variables by one unit variable).
- _*i*_ denotes an STF,
- *t* the year.
- *y* the dependent variable.
- *X* is a set of firm features.
- *n* is the number of owner/managers’ characteristics.
- *α* is the coefficient estimate (constant); and.
- *€* is the error term, representing all other factors that influence the dependent variable other than the independent variables in the study.

#### Operationalising Dependent and Independent Variables

Within this research, different measures were used as the main dependent variable to apprise the hypothetical propositions derived from both the traditional financing and behavioural theories. For the conceptual traditional model, loan acceptance/credit availability/access to finance was used as independent variables and firm growth in terms of monetary growth as dependent variables, whereas for the behavioural model, financial choices/types of financing served as dependent variables and owner managers’ personal characteristics as independent variables. Table 2 presents a summary of the indicators of the research variables.

**Table 2:** Sociodemographic profile of STF owners and their businesses

**Variables/category**	**Number**	**%**
	***N* = 140**	
*Duration of the business (years) (AB):*		
< 2	40	29
< 5	75	54
< 7	16	11
< 10	6	4
> 10	3	2
*Manpower employed (persons) (NE:)*		
1–9	38	27
10–49	62	44
50–249	30	22
More than 250	10	7
Size of business*(BS):		
Micro	65	46
Small	55	40
Medium	20	14
*Sector:*		
Hotels	50	36
Tourist agencies (agents)	55	39
International and domestic flight bookings	35	25
*Gender (G):*		
Males	110	79
Females	30	21
*Age (OA):*		
Between 20 and 30	45	28
Between 30 and 40	50	39
Between 40 and 50	37	27
Above 50	8	6
*Owners’ qualification (OQUAL):*		
Professional technical schools	21	15
Undergraduates	84	60
Master’s degree	35	25
*Entrepreneurship-related training (EET):*		
Yes	17	12
No	123	88
*Obtain financial consultation from (FC):*		
Bank manager	33	24
Accountant	35	25
Lawyer	20	14
Family	38	27
Government and support agencies (GSA)	14	10

*Categorised in consistent with the small-scale industry definition(s) based on investments

## Data Analysis and Results

### Profile of STF Owners (Entrepreneurs) and their Business

Table 2 encapsulates the sociodemographic characteristics of the sample of small tourism companies and their owner/managers used in this study. Of the total numbers of respondents, 79% were males and 21% females, with a mean age of 35 years, which shows the male dominance in terms of ownership and involvement of young people in this segment. However, there are a significant number of female employees working in this segment performing various duties, mainly as receptionists or customer care executives. Thirty-nine percent were tourist agencies and 25% international and domestic flight bookings. Eighty-three percent of small tourism businesses were set up during the last 5 years, which could be linked to the rise in the living standards (as measured by per capita income) of the middle class.

This elucidates the paradigm shift in Indian society, especially the middle class, which is educated and quite keen to travel and explore the world; consequently, the young entrepreneurs are taking the opportunity to meet those aspirations by providing different types of services. Sixty percent of the owner/managers are graduates and 25% postgraduates, which shows that education is not an issue for Indian entrepreneurs. However, entrepreneurial education (EE) is an issue. Findings suggest that STF owner/managers have low levels (12%) of entrepreneurship-related training (ERT) and hence higher needs. From the selected sample, only 15% have gained professional qualification. Hence, it can be understood that they were relying on various other sources, especially family (27%), accountants (25%), and bank managers (24%) for financial consultation. It clearly demonstrates the lack of financial decision-making skills amongst the STF owner/managers and their high reliance on external bodies. Nevertheless, lack of adequate financial education, entrepreneurial knowledge, and expertise is a foremost restraint to entrepreneurial development in developing countries like India. These results are similar with preceding studies (Sandhu, 2021; Sandhu et al., 2012a, b).

### Estimation of the Measurement Models

To test the proposed hypotheses, the statistical models were developed with covariance-based SEM (structural model estimation) methodology. Thus, the statistical models were developed with covariance-based methodology although there were no measurement errors between most of the variables. However, SEM was used to depict how psychological constructs relate to each other and influence the financing choices made by the respondents (Nachtigall et al., 2003). Therefore, confirmatory factor analysis (CFA) was used to test the validity and reliability of the research measurements, as mentioned before. Subsequently, the models were projected to examine the proposed research hypotheses, developed both for the traditional and behavioural financing models for the small tourism businesses.

The results found for the goodness-of-fit indices show the availability of the precise requirements of the measurement model. These measures included the measures of incremental fit, absolute fit, and parsimonious fit. Specifically:

- The Bentler-Bonett normed and non-normed fit index, and
- RMSEA. As such, the
- IFI,
- CFI, and
- Normed *χ*^2^ for the measurement of the parsimony of the model were also calculated.

The results confirmed that for both models:

- The RMSEA is situated in the maximum boundary (0.08),
- The normed *χ*^2^ (3.5/3.8) is under the maximum limit of 5.0, and
- The BBNFI, BBNNFI, CFI, and IFI indicators evidently surpass the suggested minimum cut-off value of 0.80 (as suggested by Hair et al. (2010) and Hu and Bentler (1999)).

**Fig. 2:**
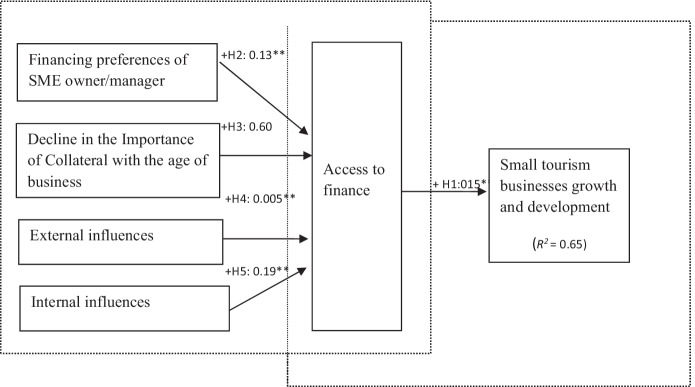
Structural model estimation (traditional conceptual model). Goodness-of-fit indices: BBNFI = 0.80; BBNNFI = 0.82; RMSEA = 0.06; CFI = 0.83; IFI = 0.83; normed *χ*^2^ = 3.80. **Standardised coefficient and *p*-value < 0.05

**Fig. 3:**
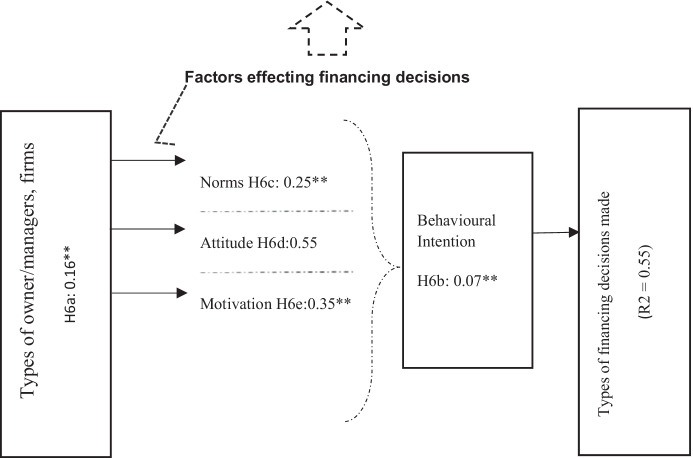
Structural model estimation (behavioural model of financing decision-making). Goodness-of-fit indices: BBNFI = 0.80; BBNNFI = 0.83; RMSEA = 0.07; CFI = 0.82; IFI = 0.82; normed *χ*^2^ = 3.50. **Standardised coefficient and *p*-value < 0.05

**Fig. 4:**
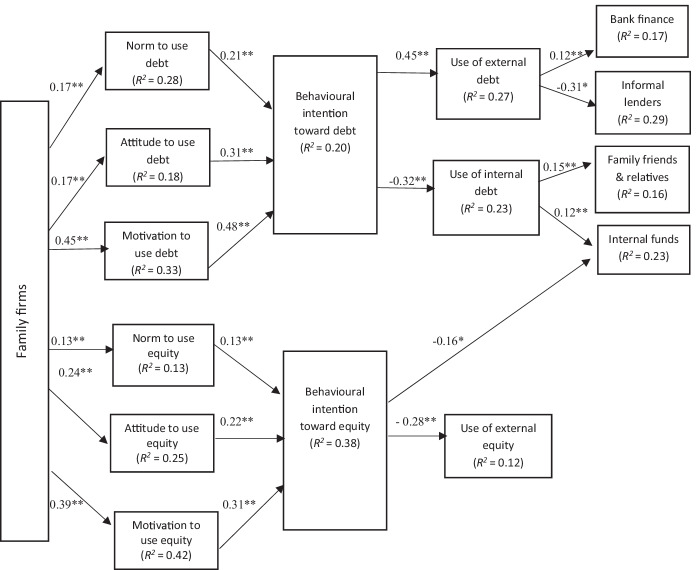
Behavioural model of financing decision-making in the family small tourism businesses. Goodness-of-fit indices: BBNFI = 0.80; BBNNFI = 0.82; RMSEA = 0.07; CFI = 0.83; IFI = 0.82; normed *χ*^2^ = 3.80. **Standardised coefficient and *p*-value < 0.05

**Fig. 5:**
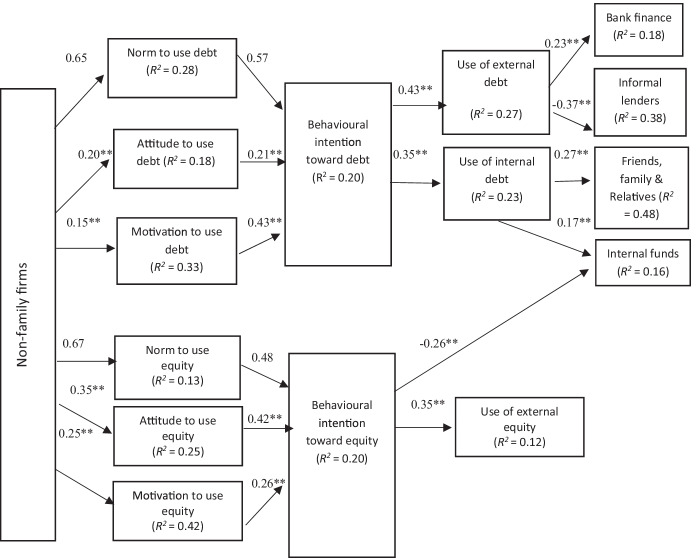
Behavioural model of financing decision making in the non-family small tourism businesses. Goodness-of-fit indices: BBNFI = 0.80; BBNNFI = 0.82; RMSEA = 0.06; CFI = 0.83; IFI = 0.81; normed *χ*^2^ = 3.50. **Standardised coefficient and *p*-value < 0.05

This suggests that these models elude the difficulties related to data non-normality and guarantee the validity of models. Figures 2, 3, 4, and 5 summarise the results of both the traditional and the behavioural financing models.

**Fig. 6:**
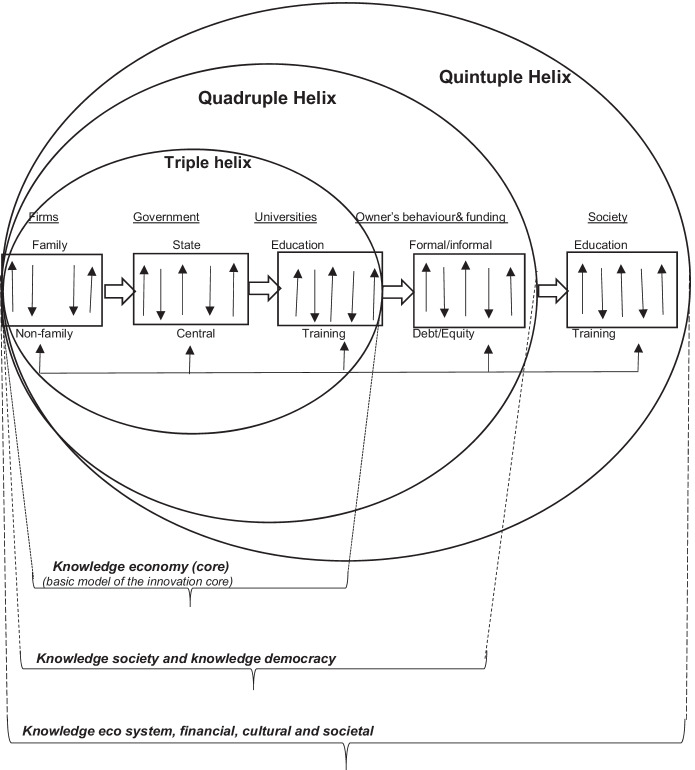
Firms, entrepreneurial orientation, access to growth finance and societal progress in an innovative financial ecosystem

#### The Conceptual Traditional Model (Fig. 2)

The hypotheses from H1 to H5 examine the influences of various variables on access to finance, growth, and development of small tourism businesses. The results confirm that access to finance (*β* = 0.15**, *p* < 0.05) has a direct effect on small tourism businesses’ growth and development, and thus, H1 is not rejected. However, changes in the financing preferences (*β* = 0.13**, *p* < 0.05) of the owner/managers have a positive impact on the obtainability of finance, so H5 is accepted. Further analysis suggests that owner/managers were using multiple sources of finance at the various stages (see Table 3). The findings suggest that owner/manager personal savings (own equity) were the most used source of finance, irrespective of the age of the business, and that external equity was only used by 5% after 10 years. As shown in Table 3, this illustrates that small tourism businesses use a good finance mix, and their use of those sources changes as the business grows.

The use of bank finance increased from 10 to 55% as the firms grew older. However, the importance of collateral did not decline with the age of the business; therefore, H2 is rejected. This is a sign that financial institutions (banks) consider SME risky clients and thus are not ready to disburse money without demanding some sort of security or collateral from them. Nevertheless, gradually with the age of the business, the proportion of bank finance exceeds funds obtained from family, friends, and even the informal lenders. Considering that, as the small tourism businesses mature, their owner/manager attain greater financial and monetary awareness and creditability; their credit history and track record now enable such firms to access formal financing. Another interesting fact emerged from the empirical analysis: those owner/managers who were from families with sound financial background and social status did not encounter any problems obtaining loans from the banks. From the selected sample, one or two incidences were reported where owner/managers even got loans without going to the bank and fulfilling all the required formalities. The reason mentioned was their social and political influence in the operational areas. However, these types of examples are difficult to find in developed countries. It shows that the competence dimension of trust positively impacts bank managers’ disposition to grant credit to small tourism businesses with a good track record, good relationship with the bank managers, sound financial background (social status/political influence), and trading history. These findings support H3 (*β* = 0.005**, *p* < 0.05) and H4 (*β* = 0.19**, *p* < 0.05).

The empirical analysis establishes the link between loan officers’ discernments of small tourism businesses and the disposition to grant them funds. Therefore, to further examine the hypothesised relationships amongst the constructs (the impact of other variables on the growth of firms and financing decisions) within the traditional conceptual model, a post hoc analysis was carried out. Tables 4 and 5 report the results of the Pearson correlation matrix and the regression model, which were conducted to further inspect the relationship among various enterprise and entrepreneurial variables to assess the magnitude of collateral and personal relationships with bank managers with respect to loan acceptance.

**Table 3:** Finance sources used by surveyed small tourism businesses at various stages

***Financial source***	***Start-up stage*** ***N* = *140***	***After 2 years***	***After five years***	***After ten years***
**Owner/managers’ personal savings**	49 (35%)	28 (20%)	28 (20%)	28 (20%)
**Bank finance**	14 (10%)	14 (10%)	35 (25%)	77 (55%)
**Borrowing from family and friends**	49 (35%)	35 (25%)	14 (10%)	7 (5%)
**Informal lenders**	28 (20%)	63 (45%)	63 (45%)	21 (15%)
**External equity**	0	0	0	7 (5%)

*N* = 140; (%) indicate the percentage of small tourism businesses in the sample using the particular financial sources

**Table 4:** Correlation matrix between different variables

***N***	**Variables**	**1**	**2**	**3**	**4**	**5**	**6**	**7**	**8**	**9**	**10**	**11**	**12**	**13**	**14**	**15**	16	17
**1**	**AB**	1																
**2**	**BS**	0.25^**^	1															
**3**	**NE**	0.32^**^	0.37^**^	1														
**4**	**GR**	0.15	0.13^**^	0.57	1													
**5**	**QUAL**	0.11	0.03	− 0.06	0.25^*^	1												
**6**	**FC**	− 0.06**	− 0.15	0.01	− 0.23**	0.07	1											
**7**	**EINRBM**	0.24^*^	0.05	0.11	0.44	0.17	0.18	1										
**8**	**IINSS**	0.054	0.09	0.16^*^	0.29^*^	0.48^*^	0.28	0.17^*^	1									
**9**	**SUPEX**	0.24	0.17^*^	0.13	0.01	0.31^*^	0.1	0.38^*^	0.05^**^	1								
**10**	**SUPIN**	0.03	0.13^*^	0.05	0.25	0.09	0.04	0.11	0.16	0.01	1							
**11**	**REQCOL**	0.15**	0.03	0.13	0.21	0.01	0.16	0.20^**^	0.32	. 38^**^	0.05	1						
**12**	**LA**	0.23^**^	− 0.08	0.26	0.09	0.07	0.02*	0.24^*^	0.34^**^	0.29^**^	0.35^**^	0.25^*^	1					
**13**	**BG**	0.49^**^	0.26**	0.27*	0.3	0.18^**^	0.25^*^	0.39**	0.23**	0.29**	0.20*	0.01	0.47**	1				
**14**	**RA**	− 0.11^**^	0.29**	0.02	0.23**	0.19*	0.13*	0.1	0.29	− 0.3	0.33	0.22	0.26*	0.08*	1			
**15**	**M**	0.13	0.82	0.13	0.31	0.20**	0.07**	0.13	0.03*	0.18*	0.15*	0.49	0.04**	0.12**	0.23*	1		
**16**	**N**	0.20**	0.35	0.30*	0.16*	− 0.37	− 0.14*	0	0.01	0.26**	− 0.12*	0.16	0.17**	0.09	− 0.02**	0.33	1	
**17**	**B**	0.03	0.13	0.12	0.27*	0.29*	0.16	0.08	0.02*	0.03*	0.43*	0.21*	0.08*	0.14*	0.27**	0.15*	0.03*	1

*N* = 140; various control variables used in post hoc analyses

*Correlation is significant at the 0.05 level (2-tailed)

**Correlation is significant at the 0.01 level (2-td)

**Table 5:** Regression coefficients

Model		Unstandardized coefficients		Standardised coefficients	T	Sig
		B	Std. error	Beta		
1^**a**^	(Constant)	1.25	0.47		2.86	0.008
	AB	− 0.10	0.03	− 0.23	− 2.98	0
	BS	0.11	0.06	0.21	1.83	0.068
	NE	0.01	0.05	0.56	0.67	0.045
	GR	0	0.08	0.66	0.05	0.98
	QUAL	0.13	0.05	0.29	2.57	0.011
	FC	− 0.02	0.03	− 0.72	− 0.77	0.375
	EINRBM	0.52	0.18	0.18	1.78	0.049
	IINSS	0.36	0.11	0.05	3.12	0.004
	SUPEX	0.19	0.09	0.19	1.98	0.054
	SUPIN	− 0.06	0.05	− 0.41	− 1.31	0.182
	REQCOL	0.35	0.25	0.07	2.63	0
	BG	0.81	0.29	0.11	5.95	0

Entire sample (*N* = 140)

*R* = 0.758; *R*-square = 0.578; Adjusted *R*-square = 0.521; standard error of the estimate = 0.295; F = 10.419; ANOVA’s test sig. = 0.000

Regression equation: *LA* = 1.25—0.10AB + 0.11BS + 0.01 + 0.00GR + 0.13QUAL—0.02FC + 0.52EINRBM + 0.36IINSS + 0.19SUPEX—0.06 + 0.35REQCOL + 0.81BG

^a^Dependent variable; LA; independent variables; AB, BS, NE, GR, QUAL, FC, EINRBM, IINSS, SUPEX, SUPIN, REQCOL, and BG

The results (Tables 4 and 5) indicated positive relationships between (i) BS and LA, (ii) QUAL and LA, (iii) EINRBM and LA, (iv) IINSS and LA, (v) SUPEX and LA, (vi) REQCOL and LA, and (vii) BG and LA. Findings propose that seven out of the 12 enterprise and entrepreneurial variables are statistically significant to the loan acceptance. The results advocate that internal influences, such as the social status of the applicant, have a strong influence on the loan acceptance with *β* = 0.05, followed by requirement of collateral (*β* = 0.07), external influences such as relationship with bank managers (*β* = 0.18) and external support (*β* = 0.19), respectively. Nevertheless, the loan acceptance was found to have a positive relation with the growth of the business (*β* = 0.11). As per Table 5, insignificant relationships between (i) NE and LA, (ii) GR and LA, (iii) FC and LA, and (iv) SUPIN and LA were found. The results suggest significant negative relationships with loan acceptance and the age of business.

As illustrated in Table 4, the age of the enterprise demonstrates a significant relationship with business size, number of employees, and relationship with bank managers. Banks understand business needs as the business grows; hence, they have a significant positive effect on the loan acceptance. The number of employees increases as the business grows. These findings are in accordance with what previous studies suggested (e.g. Sandhu et al., 2015; Sandhu & Hussain, 2015; Kumar & Ali, 2010). Moreover, a significant relationship between business size and gender was found. This was because all the females owned only micro and small businesses, while their male counterparts also ran medium enterprises along with micro and small enterprises. The findings also suggest a significant negative relationship between gender and use of a financial consultant. Most of these respondents, especially the female entrepreneurs, operated spontaneously without seeking advice or guidance when restructuring their capital mix. Inconsistently, the STF owner/managers appeared largely unacquainted of the significance of the capital structures and how such structures affect the business capital costs. Many participants acknowledged that they were short of the needed financial knowledge and that they could have profited extensively from professional guidance. These results lend some support to what was underlined by Hussain and Matlay (2007) for ethnic minority SME owner/manager in the UK. Banks provide support to the businesses as they grow, irrespective of the owner/managers’ gender. The importance of collateral was quite significant before providing loans to SMEs. Surprisingly, it was interesting to note that those owner/managers who have strong relationships with the bank managers do not need to provide collateral. The respondents indicated that good relationships with government officials were essential while applying for a loan from the bank or accessing external debt.

The findings demonstrate the relevance of the quality of financial information, importance of the input of experts (auditors, chartered accountants) while accessing bank finance. STF owner/managers revealed that while accessing bank financing, when they took help from auditors and chartered accountants to prepare all the required documentation such as financial statements, their loan application was accepted. Bank managers perceive that those small tourism businesses, which provide audited financial statements, appear more professional and authentic; hence, they are more willing to sanction and grant loans to those small tourism businesses. These excerpts from interviews with STF owner/managers signal that quality of financial information employs a positive influence on obtaining financing from financial institutions. Consequently, these audited accounts help overcome the problem of information irregularity, reduce the probability of adverse selection(s), and increased the prospects of getting bank financing. These findings support the previous research by Huguet and Gandia (2014), which emphasised the importance of audited information on the financial institutions’ lending decisions with respect to SMEs.

Some interesting results worth exploring further emerged from this research. While on the one hand banks give high priority to collateral irrespective of the gender of the respondents, on the other hand, collateral loses its importance with influential applicants. The following interview excerpt exemplifies this:

*‘I do not need to provide any documents. Bank manager knows us since last so many years. My family is well known in the area/society and we do not need to prove our financial status through the documents’.*

Many studies (Sandhu, 2021; Sandhu & Hussain, 2021; Suresh & Mohideen, 2012; Berger & Udell, 2006) have emphasised collateral as a prerequisite for accessing formal finance and the importance of relationship banking (Adel & Habib, 2018; Gobbi & Sette, 2012; Gabriel & Saurina, 2003), while none of the previous studies has portrayed that the role of discretion in relationship lending can outshine collateral due to some corrupt or malpractices and social factors.

The research results offer no support for (H2), signifying that the age of the business did not significantly reduce the impact of importance of collateral on access to finance, whereas the firms with a stronger relationship with the bank manager were less likely to provide collateral. It was supported by the above findings, as a strong positive association was found between requirement of collateral and loan acceptance, which has a substantial influence on the business growth. The findings suggest a significant influence of external (EINRBM, SUPEX) and internal factors (IINSS, SUPIN) on loan acceptance, and hence on the growth of the enterprise.

Thus, based on the above findings, hypotheses H1, H3, H4, and H5 can be supported. In contrast, hypothesis H2 can be rejected. Thus, the findings provide enormous evidence that the demand for collateral does not decline with the age of business. Banks give utmost importance to collateral and do not consider any other forms of security, irrespective of the trading activity. Hence, absence of collateral is a main restraint for SMEs when accessing formal finance from banks.

### Behavioural Model of Financing Decision-making (Figs. 3, 4, and 5)

As mentioned above, the research results propose that the financing preferences of the manager change with the age of the business. When investigated further, the empirical analysis suggests that the types of financing decisions made by STF owner/managers are influenced by what types of businesses they own (family or non-family), their behaviour, attitudes, and norms (Figs. 3, 4, and 5).

Therefore, the sixth set of hypotheses inspects the impact of small tourism corporations’ owner/managers: types, behaviour, attitudes, motivations, and norms on their financing decisions. Hypothesis H6a, which predicts that the owner/manager’s type has an impact on the financing choices made, is supported by the current data (see Fig. 3). The findings suggest that the financing choices of non-family small tourism businesses vary from those of family firms (see Figs. 4 and 5). Further investigations reveal that in the case of small tourism businesses owned by family firms, the perceived family norms have a positive influence on their attitude and behaviour intentions. Family-owned organisations are more inclined to use internal equity rather than external equity.

The resulting final behavioural models for family and non-family small tourism companies shown in Figs. 4 and 5 show standardised path coefficients and present the variance explained for each dependent variable. The results propose that family norms have greater positive influence on owner/managers’ attitudes in family firms than the non-family firms. Family norms towards external debt (*β* = 0.21**, *p* < 0.05) and external equity (*β* = 0.31**, *p* < 0.05) were found to have a significant impact on STF owner/managers’ behavioural intention towards using external debt and equity, correspondingly. However, family norms and values do not have any noteworthy impact on the behavioural intention of the owner/managers in non-family firms (which supports hypothesis H6c) (see Fig. 6).

These findings provide very thought-provoking insights about the relationship between family firms and family norms and values. Violations of these norms threaten not only the functioning of business activities, but also relationships between family members. Thus, conformity to family norms is very important for operating family-owned small tourism businesses. One of the owner/manager told us the following: ‘*For us, following our family, customs and tradition is more important than making money…. We prefer to use our internal sources rather than borrowing money externally, especially from moneylenders*’. However, during financial difficulties, we choose to borrow money from the bank rather than involving another external partner into our business, as the other person might not follow the norms and guidelines set by our family, which can ruin not only our business but will have effect on our family’s reputation. Meanwhile, when considering non-family firms, family norms do not have any influence on the small tourism businesses’ financial decision-making. When asked, one of the owner/manager replied, ‘We *have our business goals and mission. We keep our business and family norms separate from each other*.’ Grounded on the behavioural theory of the firm, the evolving solidarity in the family businesses literature is the significance of non-economic benefits that varies from business to another business based on their own priorities (Ferreira et al., 2021; Chrismas et al., 2012). These inclinations do affect their strategic decisions accordingly. It has been confirmed in literature (Chrisman & Patel, 2012) that some family corporation owners give importance to short-term concerns while others to long term. For some family members, happiness (short term) is more important, while others are highly concerned with as building a family dynasty (long term).

Furthermore, results for family and non-family organisations illustrated that an owner/manager financial attitude is considerably connected to his or her behavioural intent to use external debt financing (*β* = 0.31**, *p* < 0.05 for family firms; *β* = 0.21**, *p* < 0.05 for non-family firms) and/or external equity financing (*β* = 0.22**, *p* < 0.05 for family firms; *β* = 0.42**, *p* < 0.05 for non-family firms), thus supporting H6b and H6d. Consequently, the higher intent to use external debt increases the use of debt financing (*β* =  − 0.45**, *p* < 0.05 for family firms; *β* = 0.43**, *p* < 0.05 for non-family firms), and owner/managers’ higher intention to use external equity increases the use of external equity (*β* =  − 0.28**, *p* < 0.05 for family firms; β =  − 0.35**, *p* < 0.05 for non-family firms). Interestingly, both small tourism businesses (family and non-family) showed negative attitudes towards the use of external equity. Interestingly as well, higher intention to use external debt result in lower use of internal funds and vice versa (*β* = 0.32**, *p* < 0.05 for family firms; *β* =  − 0.35**, *p* < 0.05 for non-family firms).

Moreover, the results suggest a significant negative relationship between the behavioural intent to use bank finance (*β* = 0.12**, *p* < 0.05 for family firms; *β* = 0.23**, *p* < 0.05 for non-family firms) and informal lenders/financing in the family and non-family small tourism businesses (*β* =  − 0.31**, *p* < 0.05 for family firms; *β* =  − 0.37**, *p* < 0.05 for non-family firms). However, there is a significant relationship between owner/manager behavioural motivation and the decision to use internal funds (*β* = 0.12**, *p* < 0.05 for family firms; *β* = 0.17**, *p* < 0.05 for non-family firms). These results are in line with Ajzen’s (2002) and suggest that small tourism businesses, family-owned or non-family, partially follow pecking order theory while making financial decisions.

This evidence has been interpreted as indicating that managers’ risk preferences/attitude dominates their capital structure decisions/choices. It is not only the case with family firms, but firms in general that provide substantial attention to sustaining control and are risk-averse when their socioeconomic wealth (SEW) are endangered.

## Findings and Discussion

The analysis divulges a number of unique results. It shows that business/firm age is negatively correlated with extent of debt used by the firm. The impact of firm age on how much debt a firm uses is predominantly due to the interaction between owner/manager characteristics, behaviour, and age of business. The more supremacy that insiders retain, the less debt it uses as the firm ages. Specifically, the dynamics impelling the decision to use debt could be divergent from the factors that affect decisions on how much debt to use once the decision to use debt has been made. The informed evidence infers that the prospect of an STF using debt or equity is considerably correlated with its size, age, collateral, external and internal influences, owner/manager’s attitude, behaviour, and motivation.

The intent of this research has been to advance the understanding of the influence of owner/managers’ behavioural characteristics on their financing decision making and financial choices. Prior research (Kim et al., 2021; Owusu-Manu et al., 2021, Owusu-Manu et al., 2019, Nkwocha et al., 2019; Aninze et al., 2018; Khawaja et al., 2016; Hussain & Matlay, 2007; Hussain et al., 2006) has considered the finance gaps for SMEs and identified lack of collateral and information asymmetry as the major factors that make SME risky clients and cause them to get excluded from formal financial provisions. This paper bridges the small enterprise literature, traditional financing concepts, and extended planned behaviour theories and sets out to explain why small tourism businesses are not always innovative in using different financing sources.

From the perspective of traditional financing theories, using data on a sample of 140 small tourism businesses, the following conclusions were derived. Firstly, as the firm ages, its financial/capital structure choices differ from when the firm started. Moreover, these changes mainly explain why firm age is negatively correlated with how much formal and informal debt financing a firm uses. Secondly, an STF’s age is positively correlated with the firm’s use of debt financing. These findings are consistent with prior arguments and evidence presented by Sandhu et al. (2015) on the relationship between firm age and its use of financial leverage. However, this paper moves beyond the traditional concept to contextualise the impact of owner/managers’ behaviour on the financing choices made by them, anchoring on the theory of planned behaviour (Ajzen, 1987) to dissect the effect of owner/managers’ behaviour on financial decision-making in Indian small tourism businesses. The theory of planned behaviour recommends that an individual’s behaviour determines his/her intention, and intention predicts the tendency of the individual to engross in that behaviour.

Many other results are intriguing; for instance, conformity with family norms was found to be an important predicator in the family firms. Family-owned small tourism businesses are more expected not to use debt financing on account of norms, while non-family tourism companies are more likely not to use debt initially because of their lack of collateral and any previous past performance records, but are more likely to use debt as they age. This evidence can also be interpreted to imply that non-family firms to finance their growth, as they age, move to low-cost sources of external financing than equity financing.

There is no doubt that success of a business is a mixture of financial and non-financial factors; the psychological approach to entrepreneurship focuses on owner/manager’s personal motives, aspirations, goals, or intangible success factors when defining and measuring success. Interestingly, family-owned businesses inclined to use internal equity; however, it has been noticed that resilience of the individual played a great role not only in attracting innovative financing decision-making but also creating a novel ecosystem for their businesses to thrive in a competitive environment. Resilient owner managers moved away from the limiting beliefs/norms to use innovative sources of finance such as crowd funding, seed capital, and syndicated loan. How much they manage to avail or not? These factors demand further research investigation. Resilient owners accept change rather than resist change; consequently, they manage to seek a balance between exploration and exploitation activities in their business operations. Nevertheless, family-owned owner managers are very conscious about which network to belong in which will not negate their objectives. Consciousness of an owner/manager combined with other factors, such as culture and local networks, conscientiously create environments which enable their interactions where they think they are going to benefit and minimise conflicts.

More interesting fact emerged from the qualitative and quantitative analysis that owner managers from family or non-family firms with personality trait of autonomy to control were more aligned to exploration activities, while those with conscientiousness are closely linked with exploitation activities. Another novel finding that emerged through this research is that success of STFs operated by female owner managers are less affected by behavioural factors more by family norms and their business success remain dependent mainly on the family support where as in case of their male counterpart, overconfidence bias can have an influence and their new STF success remains dependent to their age, experience, education, social networks, and family support.

Another novelty of this research is that it not only manages to highlight the impact of psychological traits on financing choices nonetheless also manages to pull together the personality traits of the entrepreneurs.

### Research Implications

The paper has identified gaps in the empirical literature, which stem from the conventional financial decision-making theories as they failed to capture the influence of behaviour of owner managers on the financing decision-making. The motivation of the study is purely empirical rather than theoretical. It has mainly explored both an economic perspective in the form of conventional financing decision-making theories and a behavioural perspective informed by the theory of planned behaviour. How can financing choices opted by small tourism companies be understood and addressees without appreciating the owner managers behaviour? The research findings provide strong and consistent support for the postulated hypotheses and strongly emphasise the critical role of owner/managers’ behaviour in financial decision-making.

SMEs are characterised by homogeneity; currently, the main issue is certain assumptions and misconceptions made about small firms that seem not to be supported by the evidences. A thought-provoking illustration is the way in which local authorities do not distinguish between SMEs operating in different sectors, as each sector has their own unique characteristics and requirements. Therefore, when the anticipated outcomes are not informed by the SMEs from a particular sector, policymakers failed to clarify why they have failed to operate in the same manner as others. This is not just academic abandonment; however, inadequate knowledge of the needs of particular sector is a notable issue for academics working with policymakers. Consequently, for small tourism businesses and policymakers, there are clearly several policy and practical implications. Effective policy focuses on strengthening markets providing required support and funding to small tourism businesses so that they can have adequate facilities for the domestic and international tourists. The main implication for the supply side policies is that they consider small tourism companies as risky clients and do not provide them finance without asking for collateral. It will be good if the financial institutions assess individual cases and support them by considering other dynamics such as education of the owner managers, feasibility of the project, past performance of the business, enthusiasm, and passion of the owner managers. Whereas for the demand side, the current study suggests that small tourism businesses should be mindful that their behaviour inside and outside the organisation has an impact on lending choices. The conclusions drawn from this study demonstrate that many small tourism businesses are making financial decisions based on incorrect reasons, and this have led to business failure or are going to end up in disaster. Seemingly, financial education is a prerequisite for STF owner managers, to improve their access to formal finance. The primary implications for small tourism businesses are that they should consider hiring auditors or chartered accountants, establish better reputations by enhancing service quality, enhance their awareness of available financial provisions, and equip themselves with financial education. In a nutshell, this study makes several contributions from the lens of theory and policy implications. This study shows that entrepreneurship scholars need to consider a theoretical framework entailing financial, entrepreneurial, knowledge, psychological, social, and family capital to explain firms’ growth considering owner managers’ knowledge and initiatives.

## Conclusion, Limitations, and Directions for Future Research

The findings and results support the research models and most of the hypotheses. The findings helped to comprehend the effect of different behavioural/psychological traits on the financing decisions of Indian small tourism companies. Within this study, it has been found that the conventional models have failed to capture the operational attributes that can empower small tourism businesses to realise their full potential. The findings reveal that external and internal influences increase access to formal finance, the financing partialities of small tourism corporations’ owner/manager change over time, which have a positive influence on the firm growth and development. The informed evidence infers that the prospect of an STF using debt or equity is considerably correlated with its size, age, collateral, external and internal influences, owner/manager’s attitude, behaviour, and motivation. Tremendous effort and attention have been put into the planning, formulation, and development of this study. Consequently, it is anticipated that this study contributes in a substantial manner to the accumulative awareness of financing of SMEs in general and understanding factors affecting financial decision-making using theory-driven models in particular. This study clearly supports Carayannis and Campbell (2006) Quintuple Helix model of innovation ecosystem. It explicitly demonstrates that for creating innovative financing future knowledge models not only requires manifestations but coordination of main actors (businesses, financial institutions, universities/ academics, government, and policymakers) is essential (Carayannis & Campbell, 2021; Carayannis, 2019a, b, c). In the Financing Quintuple innovation system, each player plays its unique role in creating novel ecosystem and enhancing the entrepreneurial traits leading to the success of STFs.

This study has also provided directions for the future research by unveiling many avenues that remain unexplored to critical enquiry such as external and internal contacts/linkages, which play essential and diverse roles in the business lifetime. Therefore, it will be interesting to explore further why some small tourist firms achieve its objectives, while others do not.

Nevertheless, there are some limitations of this research, which need to be recognised. The first limitation of the study is that these findings cannot be generalised, as the data was collected from Delhi and Punjab. The second limitation is that this was a cross-sectional study; hence, it failed to deduce causal relationships. In order to examine these linkages more clearly, it will be good to use a longitudinal design. Third, for future research, it is imperative to provide some supplement studies using different samples to confirm the generalisability of the findings of this study internationally. To conclude, it is essential for academics/educational establishments in the arena to organise research workshops for small business owners on theories of behaviour and capital structure.

## References

[CR1] Adel, G., & Habib, A. (2018). Mediating role of entrepreneurial orientation on the relationship between relational network and competitive advantages of Tunisian contractors. *Journal of Knowledge Economy*, *9*(2), 665–679.

[CR2] Ajzen, I. (1987). Attitudes, traits and actions: Dispositional prediction of behaviour in social psychology. *Advances in Experimental Psychology,**20*(1), 1–63.

[CR3] Ajzen, I. (1991). The theory of planned behaviour. *Organizational Behaviour and Human Decision Processes,**50*(1), 179–211.

[CR4] Ajzen, I. (2002). Perceived behavioral control, self‐efficacy, locus of control, and the theory of planned behavior 1. *Journal of applied social psychology, 32*(4), 665–683.

[CR5] Ajzen, I., & Schmidt, P. (2020). Changing behaviour using the theory of planned behavior. In M. Hagger, L. Cameron, K. Hamilton, N. Hankonen, & T. Lintunen (Eds.), *The Handbook of Behavior Change* (pp. 17–31). Cambridge University Press.

[CR6] Ateljevic, J. (2007). Small tourism firms and management practices in New Zealand: The Centre Stage Macro Region. *Tourism Management,**28*(1), 307–316.

[CR7] Ang, J. (1992). On the theory of finance for privately held firms. *The Journal of Entrepreneurial Finance,**1*(3), 185.

[CR8] Aninze, F., El-Gohary, H., & Hussain, J. (2018). The role of microfinance to empower women: The case of developing countries. *International Journal of Customer Relationship Marketing and Management (IJCRMM),**9*(1), 54–78.

[CR9] Babbie, E., & Mouton, J. (2002). *The practice of social study*. Oxford University Press.

[CR10] Badulescu, D., Giurgiu, A., Istudor, N., & Badulescu, A. (2015). Rural tourism development and financing in Romania: A supply-side analysis. *Agricultural Economics,**61*(2), 72–82.

[CR11] Baixauli-Soler, J., Belda-Ruiz, M., & Sánchez-Marín, G. (2021). Socioemotional wealth and financial decisions in private family SMEs. *Journal of Business Research,**123*(1), 657–668.

[CR12] Balli, F., Curry, J., & Balli, H. (2015). Inter-regional spillover effects in New Zealand international tourism demand. *Tourism Geographies,**17*(2), 262–278.

[CR13] Barton, S., & Gordon, P. (1988). Corporate strategy and capital structure. *Strategic Management Journal,**9*(6), 623–632.

[CR14] Beck, T., Demirguc-Kunt, A., Laeven, L., & Maksimovic, V. (2006). The determinants of financing obstacles. *Journal of International Money and Finance,**25*(6), 932–952.

[CR15] Beck, T., Demirguc-Kunt, A., & Maksimovic, V. (2005). Financial and legal constraints to growth: Does firm size matter? *Journal of Finance,**60*(1), 137–177.

[CR16] Beck, T., Demirguc-Kunt, A., & Maksimovic, V. (2008). *Bank financing for SMEs around the world: Drivers, obstacles, business models, and lending practices*. Policy Research Working paper, No. 4785. Washington D.C.: World Bank. Available at https://openknowledge.worldbank.org/bitstream/handle/10986/6315/WPS4785.pdf. Accessed 1 June 2020.

[CR17] Beck, T., Demirguc-Kunt, A., & Peria, M. (2011). Bank financing for SMEs: Evidence across countries and bank ownership types. *Journal of Financial Services Research,**39*(1–2), 35–54.

[CR18] Berger, A., & Udell, G. (2006). A more complete conceptual framework for SME finance. *Journal of Banking and Finance,**30*(11), 2945–2966.

[CR19] Bridge, S., O’Neill, K., & Crombie, S. (2008). *Understanding enterprise, entrepreneurship and small business* (3rd ed.). Palgrave Macmillan.

[CR20] Britton, S. (1982). The political economy of tourism in the Third World. *Annals of Tourism Research,**9*(3), 331–358.

[CR21] Bruno, A., & Tyebjee, T. (1985). The entrepreneur’s search for capital. *Journal of Business Venturing,**1*(1), 61–74.

[CR22] Carayannis, E. (2019a). From Industry 4.0 to Industry 5.0 and the quintuple innovation helix framework – Theories, policies and practices. CI Food Webinar Series: Dr. Elias G. Carayannis Event, Teaching by Elias G. Carayannis. https://www.mcgill.ca/desautels/channels/event/ci-food-webinar-series-dr-elias-g-carayannis-296951. Accessed 25 August 2020.

[CR23] Carayannis, E. (2019b). Innovation ecosystems and artificial intelligence. Teaching by Elias G. Carayannis. https://www.iem.fraunhofer.de/de/termine/archiv/2019b/workshop-innovation-ecosystems-artificial-intelligence.html. Accessed 25 August 2020.

[CR24] Carayannis, E. (2019c). Towards industry and society 5.0. ICSB Exchange: Fall Series. Teaching by Elias G. Carayannis. https://www.youtube.com/watch?v=CEPE_vDfyv0. Accessed 25 August 2020.

[CR25] Carayannis, E., & Campbell, D. (2021). Democracy of climate and climate for democracy: The evolution of Quadruple and Quintuple Helix Innovation Systems. *Journal of Knowledge Economy,**12*(4), 2050–2082.

[CR26] Carayannis, E., & Campbell, D. (2006). “Mode 3”: Meaning and implications from a knowledge systems perspective, 1–25. In E. G. Carayannis, & D. F. J. Campbell (Eds.), *Knowledge creation, diffusion, and use in innovation networks and knowledge clusters*. *A comparative systems approach across the United States, Europe and Asia*. Westport, Connecticut: Praeger.

[CR27] Carlislie, S., Kunc, M., Jones, E., & Tiffin, S. (2013). Supporting innovation for tourism development through multi-stakeholder approaches: Experiences from Africa. *Tourism Management,**35*(1), 59–69.

[CR28] Cernat, L., & Gourdon, J. (2012). Paths to success: Benchmarking cross-country sustainable tourism. *Tourism Management,**33*(5), 1044–1056.

[CR29] Chang, S. V., Witteloostuijn, A., & Eden, L. (2010). From the editors: Common method variance in international business research. *Journal of International Business Studies,**41*(2), 178–184.

[CR30] Chou, M. (2013). Does tourism development promote economic growth in transition countries? *A Panel Data Analysis, Economic Modelling,**33*(1), 226–232.

[CR31] Chrisman, J., Chua, J., Pearson, A., & Barnett, T. (2012). Family involvement, family influence, and family-centered non-economic goals in small firms. *Entrepreneurship Theory and Practice,**36*(2), 267–293.

[CR32] Chrisman, J., & Patel, P. (2012). Variations in RandD investments of family and nonfamily firms: Behavioural agency and myopic loss aversion perspectives. *Academy of Management Journal,**55*(4), 976–997.

[CR33] Conner, M. (2020). Theory of planned behavior (4th ed.). In G. Tenenbaum, & R. C. Eklund (Eds.), *Handbook of sport psychology* (4^th^ ed., pp. 3–18). West Sussex, United Kingdom: Wiley.

[CR34] Cooper, D., & Schindler, P. (2008). *Business research methods* (10th ed.). McGraw-Hill Irwin.

[CR35] Delmar, F., & Wiklund, J. (2008). The effects of small business managers’ growth motivation on firm growth: A longitudinal study. *Entrepreneurship Theory and Practice,**32*(3), 437–457.

[CR36] Dosumu, O., Hussain, J., & El-Gohary, H. (2017). An exploratory study of the impact of government policies on the development of small and medium enterprises in developing Countries. *International Journal of Customer Relationship Marketing and Management,**8*(4), 1–26.

[CR37] Du, J., Guariglia, A., & Newman, A. (2015). Do social capital building strategies influence the financing behavior of Chinese private small and medium-sized enterprises? *Entrepreneurship Theory and Practice,**39*(3), 601–631.

[CR38] El-Gohary, H. (2020). Coronavirus and Halal tourism and hospitality industry: Is it a journey to the unknown? *Sustainability,**12*(21), 1–26.35136666

[CR39] El-Gohary, H. (2012). Factors affecting E-Marketing adoption and implementation in tourism firms: An empirical investigation of Egyptian small tourism organizations. *Tourism Management,**33*(5), 1256–1269.

[CR40] El-Gohary, H., Edward, D., & Eid, R. (2018). *Global perspectives on religious tourism and pilgrimage*. IGI Global, USA.

[CR41] El-Gohary, H., & Eid, R. (2014). *Emerging research on Islamic marketing and tourism in the global economy*. IGI Global, USA.

[CR42] El-Gohary, H., & Eid, R. (2012). DMA model: Understanding digital marketing adoption and implementation by islamic tourism organizations. *Tourism Analysis,**17*(4), 523–532.

[CR43] European Union [EU]. (2009). Enterprise and industry. [Online] Available at http://ec.europa.eu/enterprise/index-eu.htm. Accessed 9 January 2012.

[CR44] Ferreira, J., Cardim, S., & Coelho, A. (2021). Dynamic capabilities and mediating effects of innovation on the competitive advantage and firm’s performance: The moderating role of organizational learning capability. *Journal of Knowledge Economy,**12*(2), 620–644.

[CR45] Fu, X., Ridderstaat, J., & Jia, H. (2020). Are all tourism markets equal? Linkages between market-based tourism demand, quality of life, and economic development in Hong Kong. *Tourism Management*, *77*(1), 104015.

[CR46] Gabriel, J., & Saurina, J. (2003). Collateral, type of lender and relationship banking as determinants of credit risk. *Journal of Banking and Finance,**28*(9), 191–221.

[CR47] García-Villaverde, P., Elche, D., Martínez-Pérez, Á., & Ruiz-Hortega, M. (2017). Determinants of radical innovation in clustered firms of the hospitality and tourism industry. *International Journal of Hospitality Management,**61*(1), 45–58.

[CR48] Gjergji, R., Lazzarotti, V., & Visconti, F. (2022). Socioemotional wealth, entrepreneurial behaviour and open innovation breadth in family firms: The joint effect on innovation performance. Creativity and Innovation Management. Available from: https://onlinelibrary.wiley.com/doi/abs/10.1111/caim.12478. Accessed 30 January 2022.

[CR49] Glaser, B. (2004). Naturalist inquiry and grounded theory. In *Forum Qualitative Social Research*. Retrieved September 2018, from http://www.qualitative-reserach.net/fqs-texte/1-04/1-04glaser-e.htm. Accessed 12 Feb 2019.

[CR50] Gliem, J., & Gliem, R. (2003). Calculating, interpreting, and reporting cronbach’s alpha reliability coefficient for likert-type scales. Midwest Research-to-Practice Conference in Adult, Continuing, and Community Education. Available at https://scholarworks.iupui.edu/bitstream/handle/1805/344/Gliem%20%26%20Gliem.pdf?sequence=1andisAllowed=y. Accessed 5 February 2017.

[CR51] Gobbi, G., & Sette, E. (2012). Relationship lending in a financial turmoil. [Online]. Available at http://www.mofir.univpm.it/files/working%20paper/Mofir_59.pdf. Accessed 17 March 2012.

[CR52] Graham, J., & Harvey, C. (2001). The theory of practice of corporate finance: Evidence from the field. *Journal of Financial Economics,**60*(2–3), 187–243.

[CR53] Griffee, D. (2012). *An introduction to second language research methods: Design and data* (1st ed.). TESL-EJ Publications.

[CR54] Grindley, P., & Teece, D. (1997). Managing intellectual capital: Licensing and cross-licensing in semiconductors and electronics. *California Management Review,**39*(2), 8–41.

[CR55] Hair, J. F., Anderson, R. E., Babin, B. J., & Black, W. C. (2010). Multivariate data analysis: A global perspective (Vol. 7).

[CR56] Hair, J., Anderson, R., Tatham, R., & Black, W. (1998). *Multivariate data analysis* (5th ed.). Prentice Hall International.

[CR57] Hall, C., & Page, S. (2009). Progress in tourism management: From the geography of tourism to geographies of tourism–A review. *Tourism Management,**30*(1), 3–16.

[CR58] Hamad, H., Elbeltagi, I., & El-Gohary, H. (2018). An empirical investigation of business-to-business E-commerce adoption and its impact on SMEs competitive advantage: The case of Egyptian manufacturing SMEs. *Strategic Change: Briefings in Entrepreneurial Finance,**27*(3), 209–229.

[CR59] Holt, D., Madison, K., & Wu, C. (2017). Variance in family members’ assessments: The importance of dispersion modelling in family firm research. *Family Business Review,**30*(1), 61–83.

[CR60] Holmes, S., & Kent, P. (1991). An empirical analysis of the financial structure of small and large Australian manufacturing enterprises. *The Journal of Entrepreneurial Finance,**1*(2), 141.

[CR61] Hsu, D., Wiklund, J., & Cotton, R. (2017). Success, failure, and entrepreneurial re-entry: An experimental assessment of the veracity of self-efficacy and prospect theory. *Entrepreneurship Theory and Practice,**41*(1), 19–47.

[CR62] Hu, L., & Bentler, P. (1999). Cut off criteria for fit indexes in covariance structure analysis: Conventional criteria versus new alternatives. *Structural Modelling,**6*(1), 1–55.

[CR63] Huguet, C., & Gandia, J. (2014). Cost of debt capital and audit in Spanish SMEs. *Spanish Journal of Finance and Accounting,**43*(3), 266–289.

[CR64] Hussain, J., & Matlay, H. (2007). Financing preferences of ethnic minority owner/managers in the UK. *Journal of Small Business and Enterprise Development,**14*(3), 487–500.

[CR65] Hussain, J., Millman, C., & Matlay, H. (2006). SME financing in the UK and in China: A comparative perspective. *Journal of Small Business and Enterprise Development,**13*(4), 584–599.

[CR66] Hussain, J., Sandhu, N., El-Gohary, H., & Edwards, D. J. (2020). The reality of financing small tourism firms: The case of Indian tourism SMEs. *International Journal of Customer Relationship Marketing and Management,**11*(1), 64–80.

[CR67] Indian Chamber of Commerce (ICC). (2019). Tourism Available on https://www.indianchamber.org/sectors/tourism. Accessed 10 February 2019.

[CR68] Jaafar, M., Abdul-Aziz, A., Maideen, S., & Mohd, S. (2011). Entrepreneurship in the tourism industry: Issues in developing countries. *International Journal of Hospitality Management,**30*(4), 827–835.

[CR69] Kalhoro, F., Bhutto, N., Maari, S., Bibi, S., & Butt, F. (2011). Small and medium enterprises as engine of economic growth: A cross country analysis. [Online]. Available at http://saicon2011.ciitlahore.edu.pk/Economics/11-1303%20farooq%20ahmed.pdf. Accessed 7 March 2012.

[CR70] Khawaja, A., Hussain, J., & El-Gohary, H. (2016). *Bank lending to the UK and Pakistani SMEs: A survey of the literature, ISBE 2016 Conference, Institutional Voids, Entrepreneurship and Small Business Development, 27th and 28th of October 2016*. France.

[CR71] Kieschnick, R., & Moussawi, R. (2017). Firm age, corporate governance and capital structure choices. *Journal of Corporate Finance,**48*(1), 597–614.

[CR72] Kim, C. (2006). E-tourism: An innovative approach for the small and medium-sized tourism enterprises (SMTES) in Korea. In *Innovation and growth in tourism* (pp. 135–146). Paris, France: Organisation for Economic Co-operation and Development. http://www.oecd.org/dataoecd/56/13/34268048.pdf. Accessed 5 July 2021.

[CR73] Kim, K., Choi, S., & Lee, S. (2021). The effect of a financial support on firm innovation collaboration and output: Does policy work on the diverse nature of firm innovation? *Journal of Knowledge Economy,**12*(2), 645–675.

[CR74] King, S., Solomon, G., & Fernald, L., Jr. (2001). Issues in growing a family business: A strategic human resource model. *Journal of Small Business Management,**39*(1), 3–13.

[CR75] Koropp, C., Kellermans, F., Grichnik, D., & Stanley, L. (2014). Financial decision making in family firms: An adaptation of the theory of planned behaviour. *Family Business Review,**27*(4), 307–327.

[CR76] Krueger, N., Reilly, M., & Carsrud, A. (2000). Perceived difficulty in the theory if planned behaviour: Perceived behavioural control or effective attitude? *British Journal of Business Venturing,**15*(1), 411–432.

[CR77] Kuiken, A. (2015). Theory of planned behaviour and the family business. In M. Nordqvist, L. Melin, M. Waldkirch, & G. Kumeto (Eds.), *Theoretical Perspectives on Family Businesses* (pp. 99–118). Edward Elgar Publishing.

[CR78] Kumar, S., & Ali, J. (2010). Indian agri-seed industry: Understanding the entrepreneurial process. *Journal of Small Business and Enterprise Development,**17*(3), 455–474.

[CR79] Kumar, S., & Rao, P. (2016). Financing patterns of SMEs in India during 2006 to 2013- An empirical analysis. *Journal of Small Business and Entrepreneurship,**28*(2), 97–131.

[CR80] Langley, A., & Abdallah, C. (2011). Templates and turns in qualitative studies of strategy and management: Building methodological bridges. *Research Methodology in Strategy and Management,**6*(1), 201–235.

[CR81] Li, H., Chen, J., Li, G., & Goh, C. (2016). Tourism and regional income inequality: Evidence from China. *Annals of Tourism Research,**58*(1), 81–99.

[CR82] Lim, W., & Weissmann, M. (2021). Toward a theory of behavioral control. *Journal of Strategic Marketing*, 1–27. Available at 10.1080/0965254X.2021.1890190. Accessed 18 January 2022.

[CR83] Lynch, P. (1998). Female microentrepreneurs in the host family sector: Ky motivations and socio-economic variables. *International Journal of Hospitality Management,**17*(3), 379–395.

[CR84] Miller, D., & Salkind, N. (2002). *Handbook of Research Design and Social measurement*. Sage.

[CR85] Modigliani, F., & Miller, M. (1963). Corporate income taxes and the cost of capital: A correction. *American Economic Review,**1*(1), 433–443.

[CR86] Morrison, A., & Teixeira, R. (2004). Small business performance: A tourism sector focus. *Journal of Small Business and Enterprise Development,**11*(2), 166–173.

[CR87] Motta, V., & Sharma, A. (2020). Lending technologies and access to finance for SMEs in the hospitality industry. *International Journal of Hospitality Management, 86*(102371), 1–9. 10.1016/j.ijhm.2019.102371. Accessed 11 November 2020.

[CR88] Myers, S., & Majluf, N. (1984). Corporate financing and investment decisions when firms have information that investors do not have. *Journal of Financial Economics,**13*(2), 187–221.

[CR89] Nachtigall, C., Kroehne, U., Funke, F., & Steyer, R. (2003). (Why) Should we use SEM? Pros and cons of structural equation modelling. *Methods of Psychological Research, 8*(2), 1–22.

[CR90] Nemasetoni, I., & Rogerson, C. (2005). Developing small firms in township tourism: Emerging tour operators in Gauteng, South Africa. *Urban Forum,**16*(2–3), 196–213.

[CR91] Newman, A., Gunnessee, S., & Hilton, B. (2011). Applicability of financial theories of capital structure to the Chinese cultural context: A study of privately owned SMEs. *International Small Business Journal*, *30*(1), 65–83. [Online]. Available at http://isb.sagepub.com/content/early/2011/05/26/0266242610370977.full.pdf. Accessed 12 January 2012.

[CR92] Nkwocha, O. U., Hussain, J., El-Gohary, H., Edwards, D. J., & Ovia, E. (2019). Dynamics of group lending mechanism and the role of group leaders in developing countries: Evidence from Nigeria. *International Journal of Customer Relationship Marketing and Management (IJCRMM),**10*(3), 54–71.

[CR93] Ohlan, R. (2017). The relationship between tourism, financial development and economic growth in India. *Future Business Journal,**3*(1), 9–22.

[CR94] Owusu-Manu, D. G., Asiedu, R. O., Edwards, D. J., Donkor-Hyiaman, K., Abuntori, P. A., & El-Gohary, H. (2019). An assessment of mortgage loan default propensity in Ghana. *Journal of Engineering, Design and Technology*.

[CR95] Owusu-Manu, D. G., Mankata, L., Edwards, D., El-Gohary, H., & Hussain, J. (2021). A cognizance of green bond features preferential to renewable energy project financing in Ghana. IEEE Transactions on Engineering Management.

[CR96] Page, S., Forer, P., & Lawton, G. (1999). Small business development and tourism: Terra incognita? *Tourism Management,**20*(4), 435–459.

[CR97] Park, K., & Jang, S. (2018). Pecking order puzzle: Restaurant firms’ unique financing behaviours. *International Journal of Hospitality Management,**70*(1), 99–109.

[CR98] Podsakoff, P., Mackenzie, S., Lee, J., & Podsakoff, N. (2003). Common method biases in behavioural research: A critical review of the literature and recommended remedies. *Journal of Applied Psychology,**88*(5), 879. 14516251 10.1037/0021-9010.88.5.879

[CR99] Podsakoff, P. M., & Organ, D. W. (1986). Self-reports in organizational research: Problems and prospects. *Journal of Management, 12*(4), 531–544.

[CR100] Psillaki, M. (1995). Rationnement du crédit et PME: Une tentative de mise en relation. *Revue Internationale PME: Économie Et Gestion De La Petite Et Moyenne Entreprise,**8*(3–4), 67–90.

[CR101] Ramaswamy, K. (2014). Small enterprises in Indian manufacturing and inclusive growth: Search for compensatory mechanisms. [Online]. Available at http://www.igidr.ac.in/pdf/publication/WP-2014-018.pdf. Accessed 16 December 2018.

[CR102] Rayappa, M., & Arora, S. (2021). Keeping up with innovation: Perspectives into the present and the future needs of the Indian food sector. *Journal of Knowledge Economy,**12*(2), 470–488.

[CR103] Reay, T. (2014). Publishing qualitative research. *Family Business Review,**27*(1), 95–102.

[CR104] Salimullah, A. (2021). Theoretical understanding and perspective analysis of investment and development in the tourism and hospitality industry in Bangladesh. In *Tourism in Bangladesh: Investment and Development Perspectives* (pp. 27–46).

[CR105] Sánchez-Medina, J., Arteaga-Ortiz, J., Naumchik, M., & Pellejero, M. (2020). The intention to quit entrepreneurship in tourism SMEs: The effect of work addiction. *International Journal of Hospitality Management,**89*(102400), 1–12.

[CR106] Sandhu, N. (2007). An empirical investigation of financial institutions’ lending policies towards agribusiness during the post-green revolution in Punjab, India. Unpublished PhD thesis, Birmingham City University, Birmingham.

[CR107] Sandhu, N. (2021). Dynamics of banks’ lending practices to farmers in India. *Journal of Small Business and Enterprise Development,**28*(1), 102–120.

[CR108] Sandhu, N., & Hussain, J. (2021). Entrepreneurship the mediating role of finance and entrepreneurial education for small farmers in developing countries: Evidence from India. *International Journal of Entrepreneurial Behavior & Research,**27*(6), 1403–1422.

[CR109] Sandhu, N., & Hussain, J. (2015), September. Financing constraints faced by small and medium tourist businesses in India. In *European Conference on Innovation and Entrepreneurship* (p. 618). Academic Conferences International Limited.

[CR110] Sandhu, N., & Hussain, J. (2017) Financing small tourism: An Indian perspective. Paper presented at ICIE, September 2017.

[CR111] Sandhu, N., Hussain, J., & Matlay, H. (2015), February. Financing constraints and growth of SMEs in Indian Punjab. In *ICIE 2015 3rd International Conference on Innovation and Entrepreneurship*: *ICIE 2015* (p. 180). Academic Conferences Limited.

[CR112] Sandhu, N., Hussain, J., & Matlay, H. (2012a). Entrepreneurship education and training needs of family businesses operating in the agricultural sector of India. *Education + Training*, *54*(8/9), 727–743.

[CR113] Sandhu, N., Hussain, J., & Matlay, H. (2012b). Barriers to finance experienced by female owner/managers of marginal farms in India. *Journal of Small Business and Enterprise Development,**19*(4), 640–655.

[CR114] Sandhu, N., Scott, J., Gibb, J., Hussain, J., Akoorie, M., & Sinha, P. (2017). Exploring entrepreneurial finance and gender in an emergent entrepreneurial ecosystem: The case of the Punjab, northern India. *Entrepreneurial Ecosystems and Growth of Women’s Entrepreneurship: A Comparative Analysis*, 172.

[CR115] Sarria-Allende, V., Klapper, L., & Zaidi, R. (2006). Firm-level analysis of small and medium size enterprise financing in Poland. World Bank Policy Research Working Paper 3984. Washington: The World Bank.

[CR116] Schuckert, M., Kim, T., Paek, S., & Lee, G. (2018). Motivate to innovate: How authentic and transformational leaders influence employees’ psychological capital and service innovation behavior. *International Journal of Contemporary Hospitality Management,**30*(2), 776–796.

[CR117] Scandura, T., & Williams, E. (2000). Research methodology in management: Current practices, trends and implications for future research. *Academic Management Journal,**43*(6), 1248–1264.

[CR118] Schubert, S., Brida, J., & Risso, W. (2011). The impacts of international tourism demand on economic growth of small economies dependent on tourism. *Tourism Management,**32*(2), 377–385.

[CR119] Scott, S., & Bruce, R. (1994). Determinant of innovative behaviour: A path model of individual in the workplace. *Academy of Management Journal,**37*(1), 580–607.

[CR120] Shah, S., El-Gohary, H., & Hussain, J. (2015). An investigation of market orientation and tourism SMEs performance in developing countries: A review of the literature. *Journal of Travel & Tourism Marketing,**32*(8), 990–1022.

[CR121] Sharma, P., Chrisman, J., & Chua, J. (2003). Succession planning behaviour: Some empirical results. *Family Business Review,**16*(1), 1–15.

[CR122] Sharma, P., Chrisman, J., & Chua, J. (2005). Succession planning. In M. Hitt, & R. Ireland (Eds.), *The Blackwell encyclopaedic dictionary of entrepreneurship*. Oxford, UK: Blackwell.

[CR123] Shay, M. (2008). *An investigation of the attitudes, beliefs and values of elementary school teachers toward race and schooling*. University of Northern Colorado.

[CR124] Singh, S., & Turan, M. (2007). Indian tourism in the paradigm of incredible India campaign. *Journal of Hospitality Applications and Research,**2*(2), 82–101.

[CR125] Sirgy, M. (2019). Promoting quality-of-life and well-being research in hospitality and tourism. *Journal of Travel & Tourism Marketing,**36*(1), 1–13.

[CR126] Smith, S. (2006). How big? How many? Enterprise size distributions in tourism and other industries. *Journal of Travel Research,**45*, 53–58.

[CR127] Stavrov, E. (1998). A four factor model: A guide to planning next generation involvement in the family firm. *Family Business Review,**11*(2), 135–142.

[CR128] Storey, D., & Greene, F. (2010). Small business and entrepreneurship. Harlow: Financial Times/Prentice Hall.

[CR129] Storey, D. (1994). *Understanding the small business sector*. Routledge.

[CR130] Suresh, P., & Mohideen, M. (2012). Small and medium enterprise’s in India- Issues and prospects. *International Journal of Management Research and Review,**2*(2), 247–255.

[CR131] Tavakol, M., & Dennick, R. (2011). Making sense of Cronbach’s alpha. *International Journal of Medical Education,**2*, 53. 28029643 10.5116/ijme.4dfb.8dfd PMC4205511

[CR132] Thomas, R., & Augustyn, M. (2007). *Tourism in the new Europe: Perspectives on SME policies and practices* (Eds.)*.* Oxford: Pergamon*.*

[CR133] Thomas, R., Shaw, G., & Page, S. (2011). Understanding small firms in tourism: A perspective on research trends and challenges. *Tourism Management,**32*(5), 963–976.

[CR134] Tleuberdinova, A., Shayekina, Z., Salauatova, D., & Pratt, S. (2021). Macro-economic factors influencing tourism entrepreneurship: The case of Kazakhstan. *The Journal of Entrepreneurship,**30*(1), 179–209.

[CR135] Tugcu, C. (2014). Tourism and economic growth nexus revisited. A panel causality analysis for the case of the Mediterranean region. *Tourism Management*, *30*(4), 1073–1100.

[CR136] UKEssays. (2018). Analysing the tourism industry of India. [Online]. Available at https://www.ukessays.com/essays/tourism/analysing-the-tourism-industry-of-india-tourism-essay.php?vref=1. Accessed 20 November 2018.

[CR137] Weqar, F., Khan, A., & Raushan, M. (2021). Measuring the impact of intellectual capital on the financial performance of the finance sector of India. *Journal Knowledge Economy*, *12*(3), 1134–1151.

[CR138] Wu, T., & Wu, H. (2020). Causality between tourism and economic development: The case of China. *Tourism Analysis*.

[CR139] Zaki, A., El-Gohary, H., & Edwards, D. (2021). Understanding ethical and other SMEs internationalisation determinants and its impact on business performance: A primary attempt to understand Malaysian SMEs internationalisation. *International Journal of Customer Relationship Marketing and Management,**12*(1), 1–27.

[CR140] Zellweger, T., Chrisman, J., Chua, J., & Steier, L. (2019). Social structures, social relationships, and family firms. In press. [Online]. Available from http://eprints.lancs.ac.uk/127821/1/Social_structures_social_relationships_and_family_firms.pdf. Accessed 20 April 2021.

